# Effect of Cyclic Warm-Rolling Technique on Mechanical Properties of MoCu30 Thin Plates with Heterogeneous Structure

**DOI:** 10.3390/ma17163989

**Published:** 2024-08-11

**Authors:** Xianlei Hu, Huan Hu, Ruimin Lai, Qincheng Xie, Ying Zhi

**Affiliations:** 1 State Key Laboratory of Rolling and Automation, Northeastern University, Shenyang 110819, China; huxl@ral.neu.edu.cn (X.H.); 2200553@stu.neu.edu.cn (R.L.); 2170414@stu.neu.edu.cn (Q.X.); zhiying@ral.neu.edu.cn (Y.Z.); 2 Suzhou Dongbaohaixing Metal Material Science and Technology Co., Ltd., Suzhou 215625, China; 3 School of Material Science and Engineering, Northeastern University, Shenyang 110819, China

**Keywords:** cyclic warm rolling, MoCu30 alloy, amorphous interlayer, hierarchical strain band

## Abstract

By employing a cyclic warm rolling technique, MoCu30 alloy sheets of different thicknesses were prepared to investigate the effects of various rolling reduction rates on the microstructure and mechanical properties of MoCu30 alloys. Additionally, the evolution of microscale heterogeneous deformation during the tensile process was observed based on DIC technology. This study reveals that Mo–Cu interfaces at different deformation rates exhibit an amorphous interlayer of 0.5–1.0 μm thickness, which contributes to enhancing the bond strength of Mo–Cu interfaces. As the rolling reduction rate increased, the grain size of the MoCu30 alloy gradually decreased, whereas the dislocation density and hardness increased. Furthermore, the yield strength and tensile strength of the MoCu30 alloy increased gradually, whereas the elongation decreased. At a deformation rate of 74% (2 mm), the yield strength, tensile strength, and elongation of the MoCu30 alloy were 647.9 MPa, 781.8 MPa, and 11.7%, respectively. During the tensile process of Mo–Cu dual-phase heterogeneous material, a unique hierarchical strain banding was formed, which helps to suppress strain localization and prevent premature plastic instability.

## 1. Introduction

MoCu alloys are a new electronic packaging material optimized based on the research of WCu alloys, which have a lower density and are easier to deform and process than WCu alloys. Owing to the mutual incompatibility of the Cu phase and Mo, MoCu alloys are called “pseudo-alloy” [1,2,3,4]. MoCu alloys combine excellent electrical and thermal conductivity, good processing performance, and an adjustable coefficient of thermal expansion, etc., and have been widely used in the fields of electrical contact materials, electronic packaging heat sink materials, aerospace, portable instrumentation, etc. [5,6,7,8,9].

MoCu alloys have the typical characteristics of biphasic heterostructured metallic materials [10,11,12]. In recent years, heterostructured materials can effectively improve the synergistic relationship between the strength and tensile plasticity of metallic materials, which have become a new research hotspot. Heterostructured metallic materials are composite materials composed of heterogeneous regions with significant differences in mechanical properties [13,14,15], and according to the differences in the geometry and spatial arrangement of heterogeneous group elements, heterostructured metallic materials can be classified as gradient structured materials, laminar structured materials, and biphasic structured materials, etc. [16,17,18]. Gao [19] prepared heterostructured duplex steels with a martensite volume fraction above 60% using critical heat treatment, and the heterostructured duplex steels obtained using heat treatment at 780 °C and 820 °C had high strengths of 1.51 GPa and 1.74 GPa and uniform elongations of 7.6% and 4%, respectively, compared with cold-rolled ferritic pearlite steels. Fang et al. [20] prepared heterostructured duplex steels using surface mechanical grinding treatment (SMGT) to prepare gradient-nanostructured Cu, which resulted in twice the yield strength of the crude crystalline state and a uniform elongation of 31% ± 2%.

MoCu alloys are neither ductile nor brittle materials, with large differences in the properties of the Mo and Cu phases, and the deformation process is not synchronized, which makes it highly susceptible to cracks and other defects. Rolling methods, as an ideal way of plate processing, can accurately control the product size and obtain high-quality plates and plate surfaces using a low-cost and simple process. In addition, the rolling process helps to increase the densities of MoCu alloys and significantly improves the material properties. Yao et al. [21] used penetration sintering and hot-rolling processes to prepare Mo70Cu30 composites to obtain a comparison of the properties of the composites under different preparation processes. It was reported that the density of Mo70Cu30 composites increased from 98.45% to 99.79% relative to the sintered state after rolling, the tensile strength increased from 517 MPa to 753 MPa in the sintered state, and the thermal conductivity increased from 179.7 W·m^−1^·K^−1^ to 214.6 W·m^−1^·K^−1^.

In this study, 7.8 mm thick MoCu30 alloy slabs are used to prepare MoCu alloy plates with different thicknesses in combination with the cyclic warm-rolling technique, and 2 mm MoCu30 thin plates were finally obtained. The microstructure observation and mechanical property testing of MoCu alloy plates with different rolling deformation rates (unannealed) were carried out to investigate the evolution of the microstructure and mechanical properties of MoCu30 alloys during the cyclic warm-rolling process.

## 2. Materials and Methods

**Figure 1:**
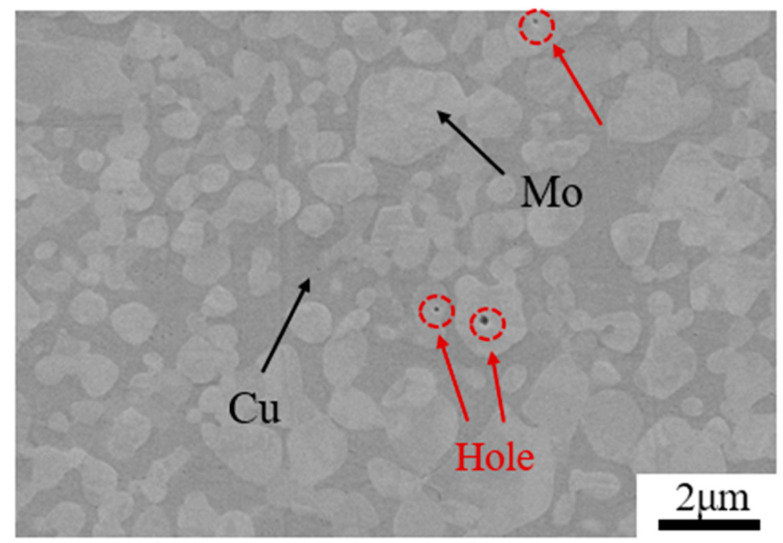
SEM image of the raw MoCu30 alloy.

**Figure 2:**
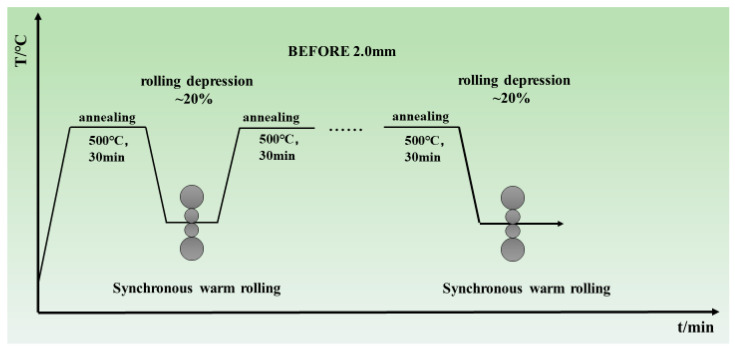
Cyclic warm-rolling process diagram.

The experimental material was a 7.8 mm thick commercial MoCu30 alloy fusion infiltration plate, and the SEM observation of the microstructure of the raw material is shown in Figure 1, in which the bright grey color indicates the Mo phase and the dark brown color represents the Cu matrix. The cyclic warm-rolling technique diagram is shown in Figure 2. The MoCu30 composite plates were subjected to multi-pass hot rolling using a conventional two-roll irreversible rolling mill at 500 °C, with multiple passes of hot rolling performed after each annealing treatment. The cumulative deformation rate per annealing was ≤20%. Samples with cumulative total deformation rates of 15%, 56%, and 74% were analyzed.

The density of the MoCu30 alloy with different reduction rates was determined via the Archimedes drainage method. The tensile strength of the alloy was determined using a CMT05 (Jinan Liangong Testing Technology Co., Ltd., Jinan, China) electronic universal testing machine. The standard reference for tensile tests at room temperature is GB/T228.1-2021 [22]. Phase identification of the samples was performed via a SmartLab (Beijing Ricochet Seth Technology Co., Ltd., Beijing, China) model X-ray diffractometer (XRD). The diffraction angle range was 10° to 90°, with a scanning speed of 5°/min. The hardness of the MoCu30 alloy was determined using a Bruker TI 950 (Bruker, Billerica, MA, USA) nanoindenter. The microstructure of the alloy was observed using a FEI Quanta 600 (Thermo Fisher Scientific, Waltham, MA, USA) model scanning electron microscope (SEM) and energy dispersive spectrometer (EDS). The micro-zone composition of the Mo and Cu junction was analyzed using a JXA-8530F (Tokyo, Japan) field emission electron probe (EPMA). The microstructure was characterized using a ZEISS GeminiSEM 460 (Zeiss Company, Oberkochen, Germany) multifunctional field emission scanning electron microscope (FE-SEM) with an acceleration voltage of 20 kV and a scanning step size of 0.08 μm. The EBSD data were analyzed and processed using AztecCrystal 2.1 software.

## 3. Results and Discussion

### 3.1. Microstructure Analysis

**Figure 3:**
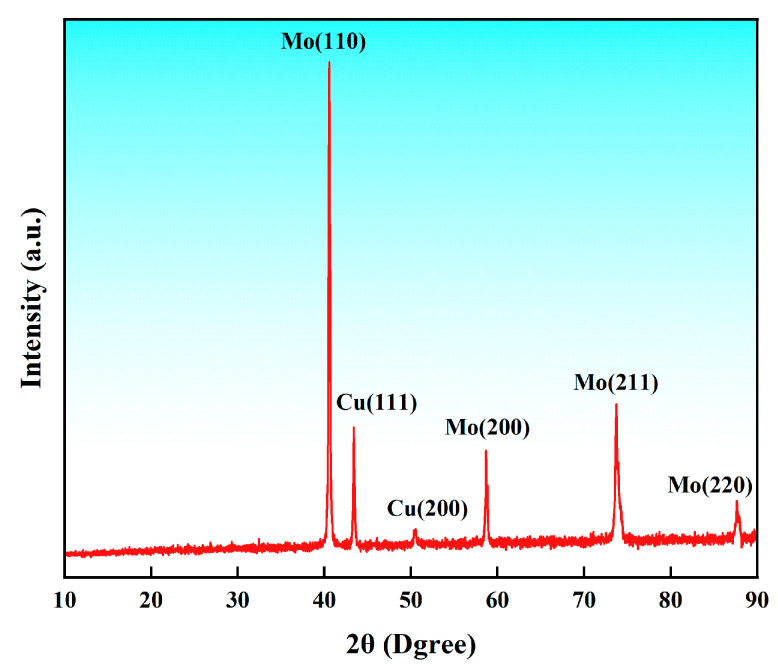
XRD pattern of the MoCu30 alloy before rolling.

Figure 3 shows the XRD pattern of the MoCu30 alloy before rolling. It can be observed that the MoCu30 alloy contains only Mo and Cu phases, with no other intermetallic compounds present.

**Figure 4:**
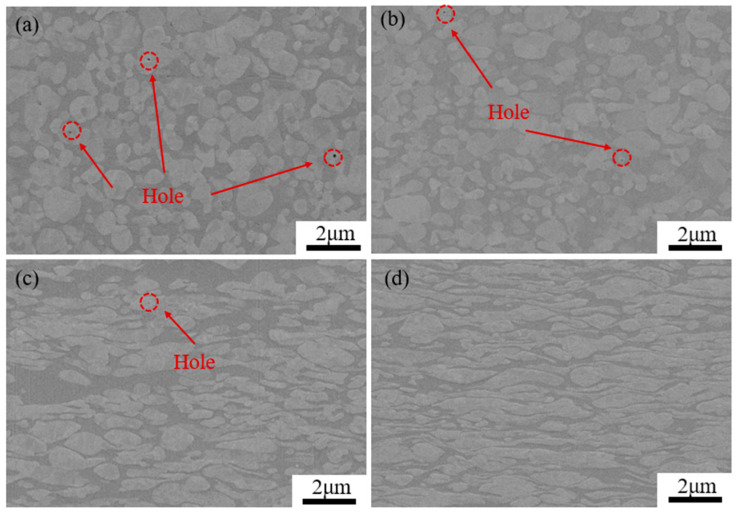
SEM images of the MoCu30 alloy with different deformation rates: (**a**) initial material; (**b**) R15%; (**c**) R56%; (**d**) R74%.

Figure 4 shows SEM images of the MoCu30 alloy with different deformation rates, in which the dark grey is the Mo phase and the grey-white is the Cu phase. Figure 4a shows that the Mo phase in the MoCu30 alloy after melting infiltration is “isometric” and adjacent to each other to form an Mo skeleton, while the Cu phase is “short sheet” distributed in the gap of the Mo skeleton. In addition, it can be observed that there are many micropores in the molten MoCu30 alloy, and most of them exist in the Mo phase. As the deformation rate increases, both the Cu and Mo phases are deformed and the micropores in the Mo phase are gradually closed. As shown in Figure 4d, under the action of rolling force, the Mo phase is gradually transformed from “equiaxial” to “cloudy”, and the Cu phase is gradually transformed from a “short flakes “to a “lamellar fibrous”, which is closely distributed around the “cloud-like” Mo phase.

**Figure 5:**
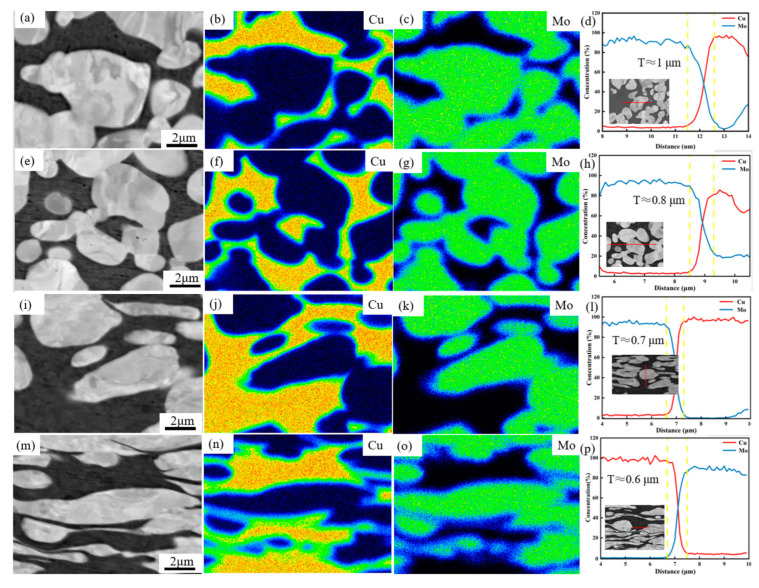
Distribution of the MoCu30 alloying elements with different deformation rates: (**a**–**d**) initial material; (**e**–**h**) R15%; (**i**–**l**) R56%; (**m**–**p**) R74%. Yellow dot lines represent the thickness of MoCu amorphous interlayer.

Figure 5 shows the elemental distribution of the MoCu30 alloys with different deformation rates. Figure 5 clearly shows that the Mo–Cu boundaries of MoCu30 alloys with different deformation rates have the presence of amorphous interlayers (short-range ordered, long-range disordered state). The thickness of the amorphous interlayers is about 0.5–1.0 μm, which is reduced by a small amount with the increase in the rolling deformation rate. The amorphous interlayer is proved to be thermodynamically stable at room temperature, and the formation of the amorphous interlayer can improve the Mo–Cu interfacial bond strength [23,24,25], which is conducive to enhancing the plasticity of MoCu alloys. In addition, compared with conventional grain boundaries, the amorphous interlayer can block the movement of dislocations more effectively and absorb the dislocations accumulated at the interface, thus acting as a toughening agent in the microstructure [26,27,28].

Table 1 presents the average grain size changes of the MoCu30 alloy with different deformation rates. It can be seen that with increasing deformation rate, the average grain size of the MoCu30 alloy decreases gradually from 6.67 μm to 2.74 μm, in which the Cu-phase grain size decreases from the initial 11.36 μm down to 1.25 μm and the Mo-phase grain size decreases from 4.59 μm down to 3.39. It is evident that the reduction in the average grain size of the Cu phase is much greater than that of the Mo phase. When the deformation rate is 74%, the Cu phase grain evolves into an ultrafine grain with a size of only 1.25 μm. The contribution of the smaller grain size to the strength is very effective.

**Figure 6:**
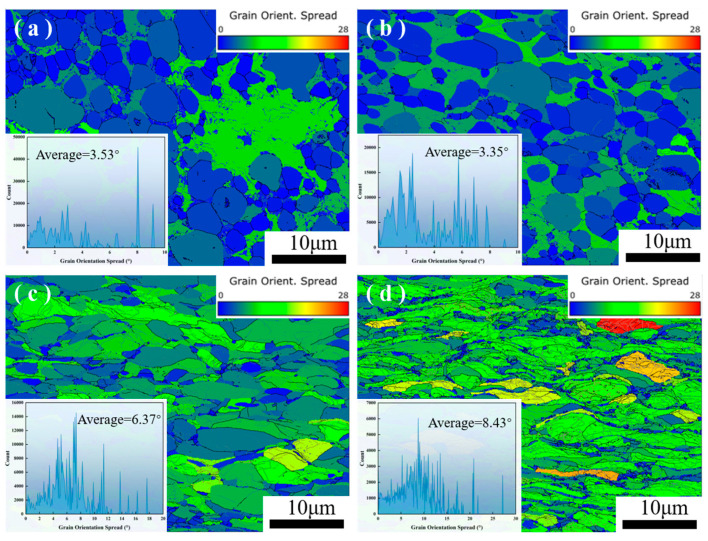
GOS of the MoCu30 alloy with different deformation rates: (**a**) initial material; (**b**) R15%; (**c**) R56%; (**d**) R74%.

Although the grain size becomes smaller with an increase in the reduction rate, the average grain orientation spread also appears to change regularly. Figure 6 shows the GOS (grain orientation spread) diagram and the average orientation spread of the MoCu30 alloy after rolling treatment with different deformation rates. It can be seen that with increasing deformation rate, the average grain orientation spread decreases and then increases. With a small deformation rate (≤15%), the internal deformation is small, the anisotropy is small, and thus the grain orientation spread decreases slightly, and the average grain orientation spread decreases from 3.53° to 3.35°. With an increasing deformation rate, the deformation of the Cu phase and the Mo phase is asynchronous, and the degree of the grain deformation is different, which leads to a large increase in the grain orientation spread, and the average grain orientation spread increases from 3.35° to 8.43°.

**Figure 7:**
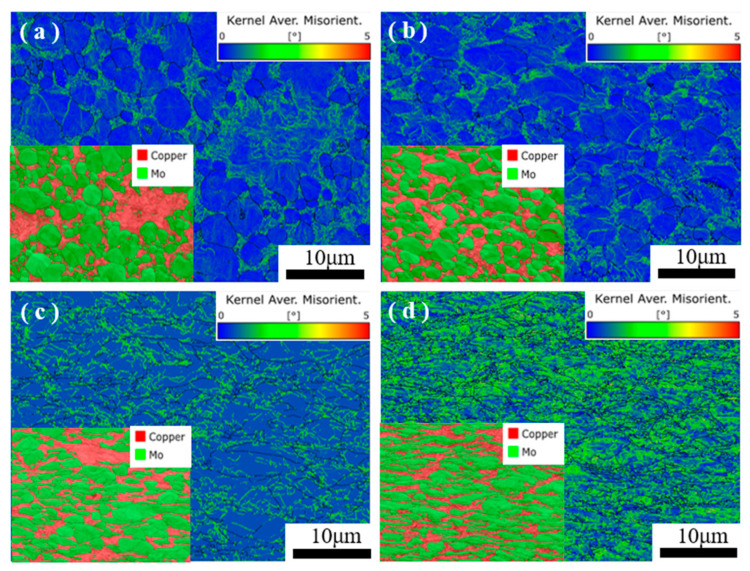
KAM plots of MoCu30 alloy with different deformation rates: (**a**) initial material; (**b**) R15%; (**c**) R56%; (**d**) R74%.

As the deformation rate increases, the grain size of the MoCu alloy becomes smaller, whereas the dislocation density at the grain boundaries and within the grains of the Cu and Mo phases gradually increase. Figure 6 shows the KAM (kernel average misorientation) plots of the MoCu30 alloy with different deformation rates. Figure 7 shows that at a 15% deformation rate, the KAM value is higher at the grain boundaries of the Cu phase, whereas the KAM value of the Mo phase is lower. When the deformation rate reaches 56%, both the interior and grain boundaries of the Cu phase show high KAM values. The KAM value at the grain boundaries of the Mo phase increases, but the interior KAM value remains low. As the deformation rate increases to 74%, the KAM value is higher at the core and grain boundaries of the Cu and Mo phases. The KAM can theoretically be used to quantify the geometrically necessary dislocation density, which responds to the degree of homogenization of plastic deformation, with higher values indicating a greater degree of plastic deformation or a higher defect density [29].

The variation in GND (geometrically necessary dislocation) density data for the MoCu30 alloys with different deformation rates is given in Table 2. The density of GNDs for both the Mo and Cu phases increase with increases in the deformation rate. Combined with Figure 7 showing the changes in the KAM values inside the MoCu alloy, it can be seen that when the deformation rate is 15%, the GNDs inside the Cu phase increase dramatically, whereas the GNDs of the Mo phase only increase by 4.1 × 10^14^ m^2^. However, when the cumulative deformation rate reaches 56%, the opposite phenomenon to the above occurs, and the GNDs inside the Mo phase begin to increase dramatically, whereas the GNDs of the Cu phase increase by 0.76 × 10^14^ m^2^ only. As the cumulative deformation rate reaches 74%, there is not much difference in the growth of GNDs between the Mo and Cu phases. This is related to the dual-phase structure of the MoCu30 alloy: Mo has a BCC structure, with high strength, high hardness characteristics, resisting plastic deformation at 500 °C, whereas Cu, with an FCC structure, is a soft-phase metal that deforms more readily. The deformation of the hard and soft phases is not synchronous during plastic deformation, which results in the formation of strain gradients near the grain boundaries [30,31,32] and the generation of GNDs that coordinate the strain gradients. When the reduction rate is low, the dislocation sources inside the soft Cu phase are the first to be activated and move toward the interface, and owing to the obstruction of the heterogeneous interfaces [33,34], the plugging accumulation is formed at the interface. The degree of dislocation plugging increases with increases in the reduction rate, and the plugged GNDs generate prestresses that act on the hard Mo phase, promoting the plastic deformation of Mo.

### 3.2. Mechanical Performance Analysis

**Figure 8:**
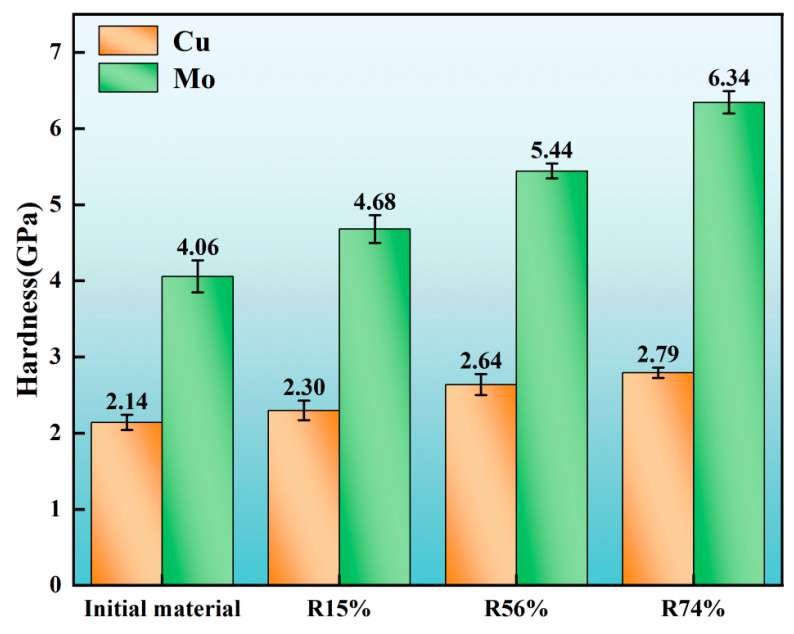
Hardness of the MoCu30 alloy with different deformation rates.

Figure 8 shows a comparison of the hardness data for the Mo and Cu phases in the MoCu30 alloy at different deformation rates. It can be seen that the hardness of both Mo and Cu phases increases with increases in the deformation rate, but the hardness of the Mo phase increases more. This is because the selected annealing temperature of 500 °C is within the range of the recrystallisation temperature of the Cu phase, whereas the recrystallisation temperature of the Mo phase (1250 °C–1450 °C) is higher, so the annealing treatment in the rolling process can effectively reduce the density of GNDs and work hardening within the Cu phase, whereas the dislocation plugging and work hardening of the Mo phase are constantly increasing, which leads to an increase in the hardness of the Mo phase at the same deformation rate much larger than that of the Cu phase.

**Figure 9:**
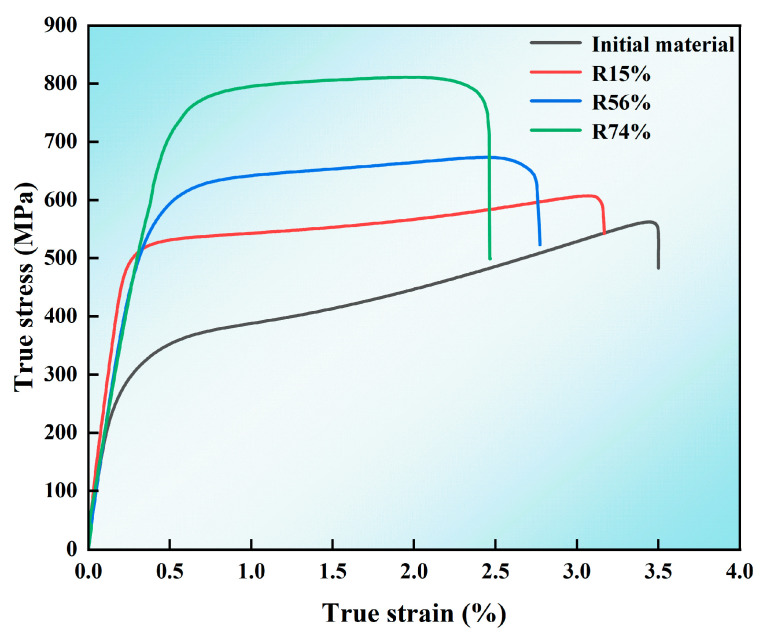
True stress–strain curve of the MoCu30 alloy with different deformation rates.

Figure 9 shows the true stress–strain curves of the MoCu30 alloys with different deformation rates. The yield strength, tensile strength, and elongation are shown in Table 3. The yield strength and tensile strength increase with the increase in the deformation rate, from 420.7 MPa and 444.2 MPa in the initial state to 647.9 MPa and 781.8 MPa, respectively. The elongation decreases with an increase in the deformation rate, from 32.2% to 11.7%. Compared to the MoCu30 alloy that was hot rolled at 850°C with a deformation rate of 80%, which had a tensile strength of 750.16 MPa and an elongation of only 2.7% [35], the samples in this study, subjected to cyclic warm rolling with a deformation rate of 74%, showed a 4.2% increase in tensile strength and a 3.33% increase in elongation.

As the deformation rate gradually increases, the Mo-Cu interfacial bond strength increases; at the same time, the grain size of Cu and Mo phases gradually decreases, the intracrystalline dislocation density increases, and the combined effect of fine-grain strengthening and work-hardening improves the strength of MoCu30 alloys [36]. However, the amorphous interlayer at the Mo-Cu interface and the weak toughening effect of the fine-grain strengthened alloy cannot make up for the plasticity reduction brought about by work-hardening, and thus the elongation of the MoCu30 alloy decreases with increasing deformation rate.

**Figure 10:**
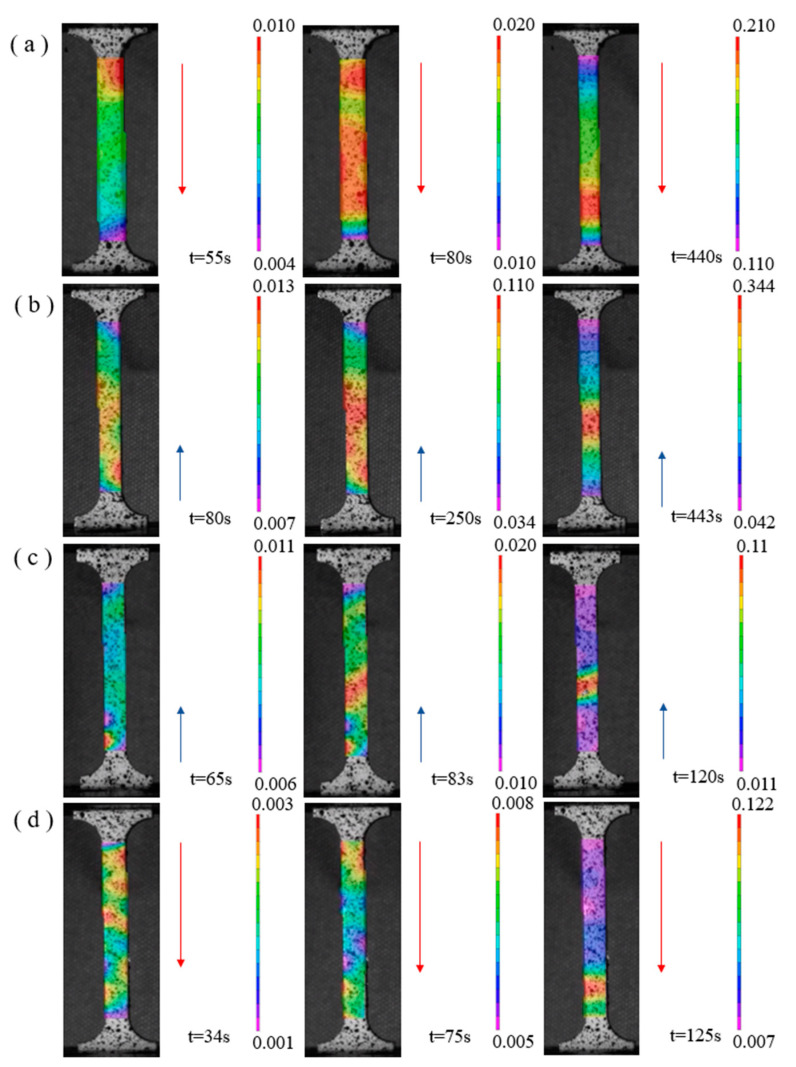
In situ observation of inhomogeneous deformation zone based on DIC technique: (**a**) initial material; (**b**) R15%; (**c**) R56%; (**d**) R74%.

Based on the DIC (digital image correlation) technique to monitor the macroscopic strain during tensile deformation of specimens with different rolling deformation rates, it was found that there were obviously discrete distributions of hierarchical strain bands during tensile deformation [37,38,39], and the angle between the strain bands and the tensile direction was mainly concentrated in the range of 30°~50°, as shown in Figure 10. Figure 10a shows that the plastic deformation starts at the upper holding end of the tensile specimen, and as the tensile continues, the hierarchical strain bands start to form at the middle and lower ends of the specimen and move downward, and finally concentrate at the lower end of the specimen, resulting in plastic instability. As shown in Figure 10, a hierarchical strain band is generated at the beginning of stretching, which gradually grows and moves upward to concentrate during the stretching process, and finally destabilizes and breaks. Figure 10c demonstrates that for a sample with a 56% deformation rate, plastic deformation initiates at the lower end of the tensile sample, and multiple strain bands progressively move upward, leading to plastic instability. Figure 10d shows that for a sample with a 74% reduction rate, multiple strain bands propagate downward before concentrating and causing instability and fracture. The formation of discrete strain bands at the stage of the plastic deformation of the MoCu30 alloy are able to uniformly apportion the plastic loading during the tensile process and inhibit the premature destabilization fracture of the material caused by the localization of strains [40]. In contrast to conventional monolithic materials in which strain localization develops to produce macroscopic strain bands that cause early fracture [41,42,43], the formation of uniformly dispersed shear bands during the stretching of heterogeneous materials is the main mechanism for adapting to the applied large plastic strains [44].

**Figure 11:**
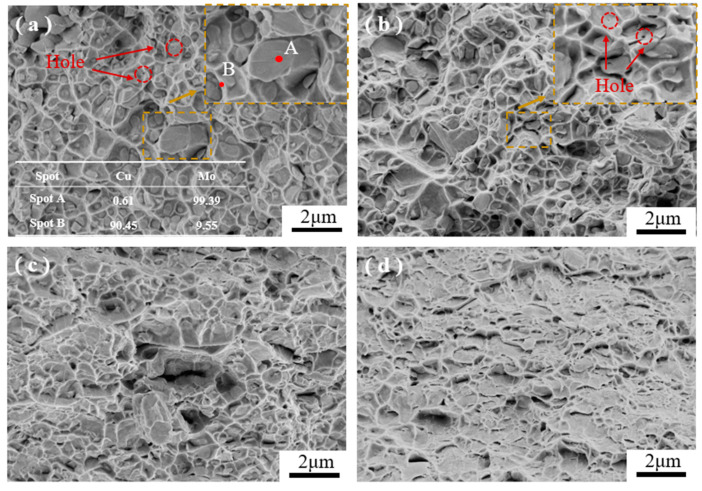
SEM–EDS diagrams of tensile fracture of the MoCu30 alloy with different deformation rates: (**a**) initial material; (**b**) R15%; (**c**) R56%; (**d**) R74%.

Figure 11 shows the SEM–EDS diagrams of the tensile fracture morphology of the MoCu30 alloy with different deformation rates. It can be seen that there are a small number of micropores within the MoCu30 alloy, and the pores gradually decrease with the increase in the rolling deformation rate, which is consistent with the SEM surface morphology observation results. Figure 11a shows that there are a large number of equiaxial ligamentous nests in the fracture of the initial material sample, and the ligamentous nests are deeper and uniformly distributed; a small number of ligamentous nests can be observed in the internal fracture of the flush and bright samples, which is a characteristic of the mixed fracture, which is tough-brittle. EDS analysis reveals that the tearing prongs on the edges of ligamentous nests are Cu, and the internal flush fracture of ligamentous nests is Mo. The Mo phase is mostly dissociated and fractured in places where the size of the Mo particles is larger and the distribution of Cu around the Mo particles is lower; in contrast, where the size of Mo phase is smaller and the distribution of the Cu phase is greater and uniform, the fracture exhibits the separation of the Mo–Cu interface and the tearing of the Cu phase [45]. Figure 11b shows that the fracture characteristics at a deformation rate of 15% are similar to those of the initial material, but the tough nest sizes are both smaller and shallower, indicating that the samples are poorly molded at a deformation rate of 15% compared to the initial material [46]. Figure 11c shows that at a deformation rate of 56%, the sample fracture begins to exhibit brittle fracture characteristics. As shown in Figure 11d, when the deformation rate is 74%, the fracture morphology exhibits a “tongue-like” pattern characteristic of quasi-cleavage brittle fracture, and the sample plasticity is also poor.

## 4. Conclusions

In this work, thin sheets of an MoCu20 alloy, 2 mm thick, were produced via cyclic warm rolling at a reduction rate of 74%. This study investigated the variation laws of grain boundary amorphous bands, grain size, and dislocation density in MoCu30 alloy plates under different deformation rates, along with corresponding mechanical property measurements. The conclusions of this study are as follows.

1. The existence of anamorphous interlayer was observed at the Mo–Cu interface of the MoCu30 alloy with different deformation rates, and the thickness of the amorphous layer decreased slightly with the increase in the rolling deformation rate. The amorphous interlayer not only enhances the bonding strength at the Mo–Cu interface, but also hinders the dislocation movement and absorbs part of the grain boundary dislocations, which plays a toughening role for the material.
2. As the rolling deformation rate increased, the average grain size of the MoCu30 alloy decreased gradually from 6.67 μm to 2.74 μm, the grain size of the Cu phase decreased from 11.36 μm to 1.25 μm initially, and the grain size of the Mo phase decreased from 4.59 μm to 3.39 μm; the average grain orientation difference of the MoCu30 alloy decreased and then increased, and the grain orientation value decreased from 3.53° to 3.35° for small deformation rates (≤15%) and the grain orientation value increased from 3.35° to 8.43° for large deformation rates (≥15%).
3. With increasing cumulative deformation during rolling, the GND density of the MoCu30 alloy increased gradually, the GND density of the Mo phase increased from the initial 2.18 × 10^14^ m^2^ to 16.01 × 10^14^ m^2^, and that of the Cu phase increased from 3.28 × 10^14^ m^2^ to 20.61 × 10^14^ m^2^, and the GND density of the Cu phase under the same deformation rate was much higher than that of the Mo phase.
4. Yield strength and tensile strength gradually increased with the increase in the rolling rate, whereas the elongation gradually decreases. At a 74% deformation rate, the yield strength of the MoCu30 alloy was 647.9 MPa, the tensile strength was 781.8 MPa, and the elongation was 11.7%. The MoCu30 alloy maintains a good elongation rate at a 74% deformation rate because of its biphasic heterogeneous structure, which forms multi-level strain bands during stretching to inhibit strain localization and prevent early plastic instability of the material. During the tensile process, a hierarchical strain band was formed to inhibit strain localization and avoid the premature plastic instability of the material.
5. During the tensile fracture process, the initial sample had internal pores and weak interfacial bonding, which manifested itself as a brittle fracture of the Mo phase and a ductile fracture of the Cu phase. The interfacial bonding strength of the MoCu30 alloy increased after rolling, and the internal micropores were reduced, but owing to the accumulation of work hardening in rolling, the strength of the material increased, the plasticity deteriorated, and there was a comprehensive performance of the brittle fracture mechanism.

## Figures and Tables

**Table 1:** Average grain size of the MoCu30 alloy with different deformation rates.

Sample	Average Grain Size (μm)	Mo Grain Size (μm)	Cu Grain Size (μm)
Initial material	6.67	4.59	11.36
R15%	5.03	6.86	4.15
R56%	3.71	4.00	3.19
R74%	2.74	3.39	1.25

**Table 2:** Variation of density data for GNDs of the MoCu30 alloys with different deformation rates.

Sample	Mo GNDs Density (×10^14^ m^2^)	Cu GNDs Density (×10^14^ m^2^)
Initial material	2.18	3.28
R15%	6.28	14.72
R56%	11.74	15.48
R74%	16.01	20.61

**Table 3:** Yield strength, tensile strength, and elongation of the MoCu30 alloys with different deformation rates.

Sample	Yield Strength (MPa)	Tensile Strength (MPa)	Elongation (%)
Initial material	420.7	444.2	32.2
R15%	517.9	534.9	25.6
R56%	557.2	632.6	14.9
R74%	647.9	781.8	11.7

## Data Availability

The original contributions presented in the study are included in the article, further inquiries can be directed to the corresponding authors.
